# A comprehensive evaluation of the role of genetic variation in follicular lymphoma survival

**DOI:** 10.1186/s12881-014-0113-6

**Published:** 2014-10-08

**Authors:** Fredrik Baecklund, Jia-Nee Foo, Paige Bracci, Hatef Darabi, Robert Karlsson, Henrik Hjalgrim, Richard Rosenquist, Hans-Olov Adami, Bengt Glimelius, Mads Melbye, Lucia Conde, Jianjun Liu, Keith Humphreys, Christine F Skibola, Karin E Smedby

**Affiliations:** Department of Medicine Solna, Clinical Epidemiology Unit, Karolinska Institutet, Stockholm, Sweden; Department of Oncology, Karolinska University Hospital Solna, Stockholm, Sweden; Human Genetics, Genome Institute of Singapore, A*STAR, Singapore, Singapore; Department of Epidemiology and Biostatistics, University of California San Francisco, San Francisco, CA USA; Department of Medical Epidemiology and Biostatistics, Karolinska Institutet, Stockholm, Sweden; Department of Epidemiology Research, Statens Serum Institut, Copenhagen, Denmark; Department of Immunology, Genetics and Pathology, Science for Life Laboratory, Uppsala University, Uppsala, Sweden; Department of Epidemiology, Harvard School of Public Health, Boston, MA USA; Department of Radiology, Oncology and Radiation Science, Uppsala University, Uppsala, Sweden; Department of Oncology and Pathology, Karolinska Institutet, Stockholm, Sweden; Division of National Health Surveillance and Research, Statens Serum Institut, Copenhagen, Denmark; Department of Epidemiology, School of Public Health, University of Alabama at Birmingham, Birmingham, AL USA; Department of Hematology, Karolinska University Hospital Solna, Stockholm, Sweden

**Keywords:** Follicular lymphoma, Prognosis, Single nucleotide polymorphism, Genome-wide association study, Candidate gene study

## Abstract

**Background:**

Survival in follicular lymphoma (FL) is highly variable, even within prognostic groups defined by tumor grade and the Follicular Lymphoma International Prognostic Index. Studies suggest that germline single nucleotide polymorphisms (SNPs) may hold prognostic information but further investigation is needed.

**Methods:**

We explored the association between SNPs and FL outcome using two approaches: 1) Two independent genome-wide association studies (GWAS) of ~300.000 SNPs followed by a meta-analysis encompassing 586 FL patients diagnosed in Denmark/Sweden 1999–2002 and in the United States 2001–2006; and 2) Investigation of 22 candidate-gene variants previously associated with FL outcome in the Danish/Swedish cohort (N = 373). We estimated time to lymphoma-specific death (approach 1 and 2) and lymphoma progression (approach 2) with hazard ratios (HR) and 95% confidence intervals (CI) in a multivariable Cox regression model.

**Results:**

In the GWAS meta-analysis, using a random effects model, no variants were associated with lymphoma-specific death at a genome-wide significant level (p < 5.0 ×10^−8^). The strongest association was observed for tightly linked SNPs on 17q24 near the *ABCA10* and *ABCA6* genes (rs10491178 HR_random_ = 3.17, 95% CI 2.09-4.79, p_random_ = 5.24 ×10^−8^). The *ABCA10* and *ABCA6* genes belong to a family of genes encoding for ABC transporter proteins, implicated in multidrug resistance. In line with a previous study, rs2466571 in *CD46* (HR = 0.73, 95% CI 0.58-0.91, p = 0.006) showed nominal association with lymphoma progression, as did two highly linked SNPs in *IL8* (rs4073 HR = 0.78, 95% CI 0.62-0.97, p = 0.02; rs2227307 HR = 0.75, 95% CI 0.60-0.94, p = 0.01) previously associated with overall survival.

**Conclusions:**

The results suggest a possible role for multidrug resistance in FL survival and add to the evidence that genetic variation in *CD46* and *IL8* may have prognostic implications in FL. Our findings need further confirmation in other independent populations or in a larger multicenter GWAS.

**Electronic supplementary material:**

The online version of this article (doi:10.1186/s12881-014-0113-6) contains supplementary material, which is available to authorized users.

## Background

For most patients with follicular lymphoma (FL) the disease is incurable and treatment aims to relieve symptoms and prolong survival [1]. In the clinical setting, established prognostic markers include tumor grade and the Follicular Lymphoma International Prognostic Index (FLIPI) [2]. However, marked variation in outcome remains within each risk group [2], and new prognostic markers are therefore desirable for more accurate prognostication and personalized treatment. In the search for such markers, several studies have assessed the impact of inherited genetic variation in FL survival [3-18].

There is growing evidence that the tumor microenvironment and the host immune response, partly determined by host genetics, are important for the clinical course of FL [19]. Germline genetic variation may further affect the metabolism [20] or receptor affinity of anti-cancer drugs [21] with possible implications for prognosis. To date, at least 30 specific germline single nucleotide polymorphisms (SNPs), mostly targeting cytokine and immune function genes (e.g., *C9*, *CD46*, *CD55*, *CFH*, *IL2*, *IL4R*, *IL8* and *FCGR2A*), have been associated with FL outcome in previous studies [3,4,6-9,11-18]. However, most studies were small in size (<150 FL cases [3,4,6,8,11-18]), and the majority of the reported findings are unconfirmed. In recent genome-wide association studies (GWAS), an important role for variation in the human leukocyte antigen (HLA) region has been revealed for *risk* of FL, and one variant in the HLA class I region has been associated both with FL risk and survival [22-26].

To gain additional knowledge of the role of genetic variation in FL outcome, we explored the association of ~300.000 SNPs with lymphoma-specific death in two independent GWAS in Sweden/Denmark [25] and the USA [22], followed by a full meta-analysis encompassing a total of 586 FL cases. We also investigated genetic variants previously reported to be associated with FL survival as well as recently established FL risk-associated SNPs for the association with lymphoma-specific death and progression in the Swedish/Danish (N = 373) and Swedish datasets (N = 231), respectively.

## Methods

This study was approved by the Ethical Review Board in each country (Sweden: The Regional Ethical Review Board in Stockholm; Denmark: Scientific Ethics Committee for the Capital Region of Copenhagen; California, USA: UCSF Human Research Protection Program, Committee on Human Subjects Research) and all study participants gave informed consent.

### SCALE study participants

We included cases with FL in the Scandinavian Lymphoma Etiology (SCALE) study, described in detail elsewhere [27]. Briefly, SCALE is a population-based case–control study of the etiology of malignant lymphomas conducted in Sweden and Denmark 1999 to 2002. Overall, 3,740 incident malignant lymphoma patients 18–74 years of age were included. Through a rapid case ascertainment network, the patients were identified shortly after diagnosis. Lymphoma subtypes were reviewed and reclassified according to the WHO classification [28]. Study participants were restricted to individuals with sufficient knowledge of the Danish or Swedish language to answer questions in a telephone interview and without a history of organ transplantation, HIV infection, or other hematopoietic malignancy. In the study overall, 85% of eligible NHL patients were included. In Sweden, a detailed registration of subtypes of all eligible patients permitted evaluation of participation rates by NHL subtype, which was 94% for FL. Early death was an uncommon reason for non-participation among the FL patients (1%). Four-hundred ninety-nine (85%) of all interviewed FL patients also gave blood. The median time from diagnosis to venipuncture was three months (interquartile range: one to five months). In SCALE overall, patients who gave blood were similar to all participating patients with regard to age, sex and educational level.

### Clinical data and follow-up

In Sweden, information about established prognostic factors (Ann Arbor stage, performance status, number of involved nodal areas, hemoglobin and lactate dehydrogenase levels), treatments and signs of progression, was collected from medical records. Lymphoma progression was defined as the time to progression, date of start of second-line treatment or lymphoma-specific death, if no second-line treatment was given (data only available in Sweden). Dates and causes of death were obtained through the Cause of Death registry [29], with complete follow-up through February 2, 2012. In Denmark, clinical parameters (Ann Arbor stage, performance status, treatment) and complete follow-up of survival through December 17, 2009, were obtained from the Danish national lymphoma database (LYFO) [30]. Lymphoma-specific death was defined as having lymphoma as the main underlying cause of death. Based on the number of FLIPI risk factors, the Swedish cases were classified as having low, intermediate or high risk of death (0–1, 2 or ≥3 risk factors, respectively). The Danish patients were classified according to a modified FLIPI based on age (≥60) and stage (I-II vs. III-IV) only, as low risk if they had none of these factors, intermediate if they had one, and high risk if they had two risk factors.

#### Genotyping

In 400 FL cases for whom a sufficient amount of DNA was available, genotyping of 317,503 SNPs was conducted at the Genome Institute of Singapore within a GWAS using the Illumina HumanHap 300 (version 1.0) array [25]. The resulting dataset was filtered on the basis of genotyping call rates (≥95%), sample completion rate (≥90%), minor allele frequency (≥0.03) and non-deviation from the Hardy-Weinberg equilibrium (p < 10^−6^). SNPs on the X or Y chromosomes or with cluster plot problems were excluded. Study participants with gender discrepancies and/or labeling errors were removed, as were samples with evidence of cryptic family relationship (identified using the genome command in PLINK [31]) and population outliers on the basis of their values of the first three principal components (identified using EIGENSTRAT software and unlinked SNPs only [32]). Complete genotyping was obtained for 298,702 SNPs in 373 (94%) of the genotyped cases.

### The University of California San Francisco (UCSF) study

The UCSF study population consisted of 213 FL cases included in a population-based case–control study of NHL (>2,055 cases, 2,081 controls) conducted in the San Francisco Bay area of incident NHL at ages 20–84 years 2001 to 2006. The cases were identified through rapid case ascertainment methods by the Cancer Prevention Institute of California with case reporting supplemented by Surveillance, Epidemiology and End Results data [33]. Overall, 69% of eligible cases participated in the study. Diagnostic material was re-reviewed and classified according to the WHO lymphoma classification by the study pathologist [28]. Blood and/or buccal specimens were collected from 87% of the cases within median 27 days from diagnosis. Genotyping of 329,294 SNPs was performed using the Illumina HumanCNV370-Duo BeadChip (Illumina, Inc., San Diego, CA). SNPs were excluded for low call rates (<90%), low minor allele frequency (<0.03), location on the sex chromosomes or cluster plot problems. Non-European samples according to MDS plot inspection were removed. Vital status and cause of death were updated through August 2012 through record linkage with the Greater Bay Area Cancer registry. Patients were censored at date of death or date of last known contact.

### Selection of SNPs associated with FL outcome and established FL risk SNPs

Published studies of germline polymorphisms and FL outcome were found by searching PubMed and Web of Science (through June 30, 2013). We selected SNPs statistically significantly (p < 0.05) associated with overall or lymphoma-specific survival or with time to progression/treatment in at least one study and investigated the association of these with lymphoma-specific death and progression in SCALE and the Swedish cohort, respectively. Fifteen studies and 30 SNPs representing 29 separate loci were identified (Table 1). Genotype data on 22 of these markers (21 loci) were available through genotyping (N = 9) or imputation (N = 13). We used 1000 genomes multi-ethnic reference panel and Impute 2 for imputation (http://mathgen.stats.ox.ac.uk/impute/impute_v2.html#download_reference_data). A strict threshold was set to genotypes with probabilities >0.9, SNPs with information scores >0.8 and call rates >0.9. Six SNPs strongly associated with FL risk in recent GWAS [22-26], of which one (rs6457327 in the HLA class II region) also has been associated with FL prognosis in two studies [4,18], were also selected for survival analysis.

**Table 1:** **List of single nucleotide polymorphisms (SNPs) associated with follicular lymphoma outcome (p < 0.05) in at least one previously published study, and results for these variants in other previous studies**

**Chr**	**Gene**	**SNP**	**Minor/major allele**	**Study**	**# FL cases**	**# SNPs tested**	**Outcome**	**Relative risk ratio**	**# Studies reporting no association [refs]**
1	C1qA	rs172378	A/G	Racilia 2008	133	3	PFS	2.5 (2.0, 3.1)	none
1	CD46	rs2466571	C/A	Charbonneau 2012	107	167	EFS	1.49 (0.86, 2.61), p_trend_ < 0.05	none
1	CD55	rs2564978	T/C	Charbonneau 2012	107	167	EFS	0.52 (0.30, 0.88)	none
1	CFH	rs1065489	T/G	Charbonneau 2012	107	167	EFS	0.44 (0.24, 0.81)	none
1	CFH	rs1329423	C/T	Charbonneau 2012	107	167	EFS	0.49 (0.29, 0.82)	none
1	CFH	rs3766404	G/A	Charbonneau 2012	107	167	EFS	2.25 (1.31, 3.87)	none
1	CFHR1	rs436719^A^	C/A	Charbonneau 2012	107	167	EFS	0.57 (0.34, 0.96)	none
1	CFHR5	rs6694672	G/T	Charbonneau 2012	107	167	EFS	2.63 (1.41, 4.92)	none
1	FCGR2A	rs1801274	A/G	Weng 2003	87	2	PFS	Decreased rate, p_log-rank_ < 0.02	4 [10,13,14,16]
			A/G	Cerhan 2007	278	73	OS	0.58 (0.31, 1.04), p_trend_ = 0.04	2 [10,13]
1	FCGR3A	rs396991^A^	G/T	Weng 2003	87	2	PFS	Decreased rate, p_log-rank_ < 0.03	5 [6,10,13,14,16]
			G/T	Ghielmini 2005	185	1	EFS	0.5 (0.3, 0.9)	1 [5]
			G/T	Persky 2012	142	2	OS	0.33 (0.11; 0.96)	1 [10]
			G/T	Cartron 2002	49	2	RR	1.5 (1.2, 1.9)	1 [10]
1	MTHFR	rs1801131	C/A	Wang 2009	192	66	OS	2.00 (1.04, 3.84)	none
1	SELE	rs5361		Aschenbrook- Kilfoy 2012	117	82	OS	0.10 (0.02, 0.48)	1 [7]
2	IL1RN	rs454078	T/A	Cerhan 2007	278	73	OS	0.50 (0.28, 0.87)	1 [3]
3	CCR2	rs1799864^A^		Aschenbrook- Kilfoy 2012	117	82	OS	0.27 (0.08, 0.86)	1 [7]
3	FTHFD	rs1127717	G/A	Wang 2009	192	66	OS	1.99 (1.07, 3.7)	none
4	IL2	rs2069762	G/T	Cerhan 2007	278	73	OS	1.81 (1.06, 3.07)	none
4	IL8	rs4073	T/A	Aschenbrook- Kilfoy 2012	117	82	OS	2.60 (1.10, 6.15)	none
			A/T	Cerhan 2007	278	73	OS	0.47 (0.27, 0.79), p_trend_ = 0.06	none
4	IL8	rs2227307	T/G	Aschenbrook- Kilfoy 2012	117	82	OS	2.57 (1.07, 6.17)	none
			G/T	Cerhan 2007	278	73	OS	0.53 (0.31, 0.91), p_trend_ = 0.11	none
4	IRF2	rs3775567^A^	T/	Gibson 2012^A^	244	6679	OS	3.18 (1.72, 5.87)	none
5	C9	rs1421094	A/G	Charbonneau 2012	107	167	EFS	0.54 (0.32, 0.90)	none
5	IL12B	rs3212227	C/A	Cerhan 2007	278	73	OS	2.01 (1.18, 3.42)	none
6	C6orf15	rs6457327	A/C	Wrench 2011	218	2	TTT	2.25. (1.16, 4.36)	none
				Berglund 2011	102	1	OS	Increased rate, p = 0.006	none
6	IFNGR1	rs3799488		Aschenbrook- Kilfoy 2012	117	82	OS	3.19 (1.09, 9.34)	1 [7]
8	GGH	rs719235	T/G	Wang 2009	192	66	OS	2.49 (1.21, 5.14)	none
9	GALNT12	rs10987898^A^	G/	Gibson 2012^A^	244	6679	OS	0.48 (0.27, 0.83)	none
9	GALNT12	rs10819377	T/	Gibson 2012^A^	244	6679	OS	0.62 (0.36, 1.03), p_trend_ < 0.001	none
11	CXCR5	rs1790192	G/A	Charbonneau 2013^A^	172	10	EFS	0.64 (0.47; 0.87)	none
16	IL4R	rs1801275		Aschenbrook- Kilfoy 2012	117	82	OS	2.35 (1.07, 5.19)	none
20	BMP7	rs6025446^A^	G/A	Gibson 2012^A^	244	6679	OS	0.41 (0.21, 0.69)	none
22	MIF	rs755622		Aschenbrook- Kilfoy 2012	117	82	OS	2.45 (1.09,5.47)	none

EFS = event-free survival, PFS = progression-free survival, OS = overall survival, TTT = time to transformation, RR = response to Rituximab. R = Rituximab, KM = Kaplan-Meier curve. ^A^Variant was not available for evaluation in the present study.

### Statistical analysis

#### Genome-wide association studies

In SCALE and UCSF separately, we estimated the association of SNPs with lymphoma-specific death using the Cox proportional hazards model. Time was measured from diagnosis to lymphoma-specific death or end of follow-up. Deaths from other causes than lymphoma were censored. Patients without an event were censored at the time of last known follow-up. The SNPs were coded as 0, 1 or 2 based on the number of minor alleles, and treated as continuous variables in the model. Adjustment was made for age at diagnosis, sex and population stratification (using the first three dimensions calculated by using EIGENSTRAT [32] in SCALE and multidimensional scaling [31] in UCSF; in both cohorts unlinked SNPs only were used for these calculations). Data on FLIPI and first-line rituximab was available in SCALE and the Swedish cohort, respectively, but not in UCSF, and hence these covariates were not included in the model. A full meta-analysis of the GWAS results was performed using the DerSimonian-Laird random effects method [34]. PLINK v 1.07 (http://pngu.mgh.harvard.edu/purcell/plink/) [31] and R [35] were used for these analyses. In complementary analyses, top variants in the meta-analysis were also adjusted for FLIPI risk groups (in SCALE) and first-line rituximab (in the Swedish cohort). The top locus in the meta-analysis was further explored in a regional association plot, combining the p-values of association in the meta-analysis, LD data from 1000 Genomes European population, gene information from the UCSC browser, and estimated recombination rates (http://csg.sph.umich.edu/locuszoom/) [36]. The potential functionality of the top SNPs and SNPs in strong LD (r^2^ ≥ 0.8) with these in the 1000 Genomes European population were explored using RegulomeDB, which integrates multiple types of functional data generated by ENCODE and other sources (http://RegulomeDB.org/) [37].

#### Investigation of SNPs with reported prognostic impact in FL and established FL risk SNPs

We investigated the association of the selected candidate SNPs and established FL risk SNPs with lymphoma-specific death in SCALE and lymphoma progression in SCALE Sweden using the same Cox model as in the GWAS. The proportional hazard assumption was tested by plotting Schoenfeld residuals against follow-up time; no violations were found. In complementary analyses, associated variants were also adjusted for FLIPI risk groups and first-line rituximab. Also, potential immortal time bias was assessed in the Swedish cohort by starting follow-up at date of venipuncture instead of diagnosis. We further applied the multi-SNP modeling of risk alleles performed in three previous studies (rs5361, rs3799488, rs1799864 and rs1800796 [3]; rs4073, rs2069762, rs3212227 and rs454078 [7]; rs1801131, rs1127717 and rs719235 [15]) on the SCALE dataset. The SNPs were then coded as 0 or 1, based on the presence of the risk (protective/deleterious) allele [3,7,15]. The impact of having one, two or three or more as compared to no risk alleles on lymphoma-specific death was assessed in a Cox model, adjusting for age and sex. Multiple testing was adjusted for with the Bonferroni method [38]. The analyses were done in SAS 9.2. Power calculations were performed in R (using survSNP) [35].

## Results

### Patient characteristics

The Danish-Swedish FL cohort was followed for a median of 8.9 years from diagnosis (range 4.3 months to 12.1 years). Median age at diagnosis was 57 years and 65% had Ann Arbor stage III or IV disease (Table 2). One-hundred thirty-seven patients (37%) died of which 88 deaths (64%) were classified as lymphoma-specific. The UCSF cohort was followed for a median of 7.5 years (range 6.9 months to 10.3 years). Twenty-six percent (N = 56) died, of which 43% (N = 24) were classified as lymphoma-specific deaths. In Swedish patients, progression occurred in 67% (N = 155), and rituximab was used in first-line treatment in 10% and overall in 47%. Overall survival differed as expected between risk groups defined by FLIPI (low, intermediate, high; p_log-rank_ < 0.05) in the total SCALE study population and in the Swedish and Danish populations separately (data not shown).

**Table 2:** **Characteristics of the study participants with follicular lymphoma in the Scandinavian Lymphoma Etiology (SCALE) study (Denmark/Sweden) and in the University of California San Francisco (UCSF) study**

	**SCALE overall N (%)**	**Swedish FL cases N (%)**	**Danish FL cases N (%)**	**UCSF FL cases N (%)**
**Number of participants**	373	231 (62)	142 (38)	213
**Sex** (men)	185 (50)	111 (48)	74 (52)	110 (52)
**Age** (years)				
18-34	9 (2)	6 (3)	2 (1)	3 (1)
35-44	30 (8)	15 (6)	13 (9)	15 (7)
45-54	105 (27)	58 (25)	43 (30)	50 (23)
55-64	139 (36)	83 (36)	53 (37)	73 (34)
65-75	100 (26)	69 (30)	31 (22)	42 (20)
76-84	-	-	-	30 (14)
Median (range)	57 (22–74)	58 (22–74)	56 (33–72)	60 (29–84)
**Participant born in Sweden or Denmark (yes)**	342 (92)	203 (88)	139 (98)	-
**Parents born in Sweden or Denmark (yes)**	331 (89)	193 (84)	138 (97)	-
**Participants of European ancestry** ^**B**^	-	-	-	213 (100)
**Ann Arbor stage**				
I	77 (22)	50 (23)	27 (19)	-
II	48 (13)	32 (15)	16 (12)	-
III	100 (28)	69 (32)	31 (22)	-
IV	133 (37)	68 (31)	65 (47)	-
Missing	15	13	2	-
**FLIPI** ^**A**^				
Low risk	136 (36)	107 (46)	29 (20)	-
Intermediate risk	152 (41)	68 (29)	84 (59)	-
High risk	85 (23)	56 (24)	29 (20)	-
**Progression (yes)**	-	155 (67)	-	-
Missing	-	2 (1)	-	-
**Transformation (yes)**	-	56 (26)	-	-
Missing	-	12 (5)	-	-
**Rituximab first line/any treatment line (yes)**	-	24 (10)/106 (47)	-	-
Missing	-	5 (2)	-	-
**Died**	137 (37)	88 (38)	49 (35)	56 (26)
**Died due to lymphoma**	88 (24)	54 (23)	34 (24)	24 (11)

^A^The Swedish patients were classified according to the number of FLIPI risk factors. The Danish patients were classified according to a modified FLIPI, based on age and stage only.

^B^Non-European samples according to MDS plot inspection were removed in UCSF.

### Genome-wide association studies and meta-analysis

In SCALE and UCSF separately, no variants were associated with lymphoma-specific death at a genome-wide significant level (p ≤ 5.0 × 10^−8^). Thirteen variants in SCALE (on chromosomes 1, 4, 8 and 17; Additional file 1: Figure S1a) and five variants in UCSF (on chromosomes 1, 2, 6, 9 and 20; Additional file 1: Figure S1b) were associated with lymphoma-specific death with a p ≤ 10^−6^, which was more than would be expected by chance (Additional file 1: Figure S2a and S2b).

In the meta-analysis of the 298,702 variants in SCALE and 319,693 in UCSF, 295,134 variants were present in both cohorts. No variant reached genome-wide significant level of association (p < 5.0 × 10^−8^). The strongest signals were observed for SNPs on chromosome 1, 4, 17 and 19 (Additional fle 1: Figure S3), for which the p-values were smaller than would be expected by chance (Additional file 1: Figure S4). The strongest association with lymphoma-specific death was observed at rs10491178 (HR_random_ = 3.17, 95% CI 2.09-4.79, p_random_ = 5.24 × 10^−8^), located on 17q24 in the ATP binding cassette A10 gene (*ABCA10*) (Table 3). An additional five SNPs on 17q24, close to *ABCA6*, showed similar associations and were in strong linkage disequilibrium (LD) with rs10491178 (r^2^ ≥ 0.96 in the SCALE cohort; Additional file 1: Figure S5). One SNP on chromosome 1 showed a suggestive association of p_random_ = 10^−7^ (rs3131729 HR_random_ = 2.45, 95% CI 1.75-3.44, p_random_ = 2.22 × 10^−7^; Table 3). Another SNP on chromosome 1 showed similar association and was linked with rs3131729 (r^2^ = 0.7) (Additional file 1: Table S1). One intergenic variant on chromosome 4 (rs11932201 HR_random_ = 2.16, 95% CI 1.57-2.97, p_random_ = 2.24 × 10^−6^) and one variant located on 19q13 (rs2250066 HR_random_ = 2.11, 95% CI 1.52-2.94, p_random_ = 9.38 ×10^−6^) also showed suggestive associations at p_random_ = 10^−6^ (Table 3). For these ten top SNPs, there was no indication of heterogeneity between the studies (p_heterogeneity_ > 0.05, I^2^ = 0; Additional file 1: Table S1).

**Table 3:** **Top single-nucleotide polymorphisms (SNPs) associated with lymphoma-specific death** ^**A**^ **(p**_**random**_ **≤ 10** ^**−6**^ **) in the meta-analysis of FL patients in SCALE and UCSF (N = 586)**

						**SCALE**		**UCSF**		**Meta-analysis**		**Heterogeneity**				
**Chr**	**SNP**	**Position**	**A1**	**A2**	**MAF**	**HR (95% CI)**	**p**_**SCALE**_	**HR (95% CI)**	**p**_**UCSF**_	**HR (95% CI)**	**p**_**RANDOM**_	**p**_**HET**_	**I** ^**2**^	**Gene**	**Left gene**	**Right gene**
17	rs10491178	64661568	A	G	0.06	3.10 (1.97; 4.89)	1.13E-06	3.50 (1.28; 9.53)	1.36E-02	3.17 (2.09; 4.79)	5.24E-08	0.83	0	ABCA10	ABCA6	LOC100133319
1	rs3131729	57971355	A	G	0.13	2.58 (1.77; 3.76)	9.24E-07	2.00 (0.92; 4.31)	7.58E-02	2.45 (1.75; 3.44)	2.22E-07	0.56	0	DAB1	C8B	LOC729423
4	rs11932201	14304530	C	A	0.17	2.10 (1.48; 2.97)	2.93E-05	2.50 (1.13; 5.55)	2.29E-02	2.16 (1.57; 2.97)	2.24E-06	0.69	0	NA	LOC152742	LOC441009
19	rs2250066	56220931	A	G	0.15	2.07 (1.45; 2.97)	7.31E-05	2.35 (1.01; 5.44)	4.40E-02	2.11 (1.52; 2.94)	9.38E-06	0.79	0	KLK11	KLK10	KLK12

A2 = major allele, MAF = minor allele frequency, SCALE = Scandinavian lymphoma etiology study, UCSF = University of California, San Francisco, NA = not applicable.

^A^Estimated with Hazard ratio, HR, and 95% confidence interval, CI, adjusting for age at diagnosis and three principal components.

^B^MAF calculated in SCALE.

Results are sorted by random effects p-value. The minor allele (A1) was investigated for association.

Among the top 48 SNPs in the UCSF GWAS with p ≤ 10^−5^ seven were not genotyped in SCALE. We were able to impute six of these with high confidence (information score >0.8). The meta-analysis of these SNPs gave no additional suggestive associations (data not shown).

There was no correlation between rs10491178 and performance status, lactate dehydrogenase level (Swedish cohort, N = 231) or FLIPI risk groups (SCALE cohort, N = 373; Fisher’s Exact test p ≥ 0.15; data not shown). For rs10491178, rs3131729, rs11932201 and rs2250066, additional adjustment for FLIPI and first-line rituximab in SCALE and the Swedish cohort, respectively, did not alter the results meaningfully (data not shown). The association of the top SNP at 17q24 with overall survival (SCALE) was weaker compared with lymphoma-specific death (rs10491178 HR = 2.00, 95% CI 1.35-2.97, p = 5.68 × 10^−4^), and there was no association with lymphoma progression (Swedish cohort; Additional file 1: Table S2).

Combining data in the RegulomeDB suggested a putative functional role for six of the ten top SNPs, of which rs10491178 (chr 17), rs11932201 (chr 4) and rs2250066 (chr 19) had RegulomeDB scores of 5, indicating that they may affect transcription factor binding or are located in a DNAse hypersensitivity site. There was no data for rs3131729 (chr 1). The lowest RegulomeDB score (i.e. strongest evidence of being in a regulatory site) among our top SNPs and highly linked variants (r^2^ ≥ 0.8) was found at the SNP rs113464685 in *ABCA10* (score 3b), in LD with rs10491178 (data not shown). After imputing the genotypes for rs113464685 in SCALE (as described in Methods for the candidate gene SNPs), we ran the Cox regression for this SNP and found estimates for lymphoma-specific death virtually identical to those of the other SNPs on 17q24 (HR = 3.10, 95% CI 1.98-4.89, p = 1.12 × 10^−6^).

### Investigation of SNPs with reported prognostic impact in FL and established FL risk SNPs

For lymphoma-specific death (SCALE cohort, N = 373), we had ≥80% power to detect HR ≥ 2.0 (or ≤0.5) for rare variants (minor allele frequency (MAF) =0.09 [8]) and HR ≥ 1.5 (or ≤0.67) for more common variants (MAF = 0.49) (Additionl file 1: Figure S6). For lymphoma progression (Swedish cohort, N = 231), we had ≥80% power to detect an HR ≥ 1.75 (or ≤0.57) for variants with MAF ≥0.09. Among the 22 candidate SNPs evaluated, two imputed variants showed nominally significant association (p < 0.05) with lymphoma-specific death: rs1801131 in *MTHFR* (HR = 0.69, 95% CI 0.49-0.97, p = 0.03) and rs2069762 in *IL2* (HR = 0.63, 95% CI 0.43-0.92, p = 0.02; Table 4). rs1801131 was also associated with lymphoma progression (HR = 0.59, 95% CI 0.45-0.77, p = 0.0001). The direction of association for rs1801131 and rs2069762 with the two lymphoma-specific outcomes was opposite of what was previously reported for overall survival (Table 1) [7,15]. In line with a previous study investigating event-free survival in FL [8], we found that the C allele of rs2466571 in the *CD46* gene was associated with shorter time to lymphoma progression (HR = 1.37, 95% CI 1.10-1.72, p = 0.006; Table 4). Likewise, the estimates for two imputed tightly linked SNPs (r^2^ = 0.99) in the *IL8* gene*,* rs4073 and rs2227307 (HR_rs4073_ = 0.78, 95% CI 0.62-0.97, p = 0.02; HR_rs2227307_ = 0.75, 95% CI 0.60-0.94, p = 0.01), and lymphoma progression were in the same direction as in two previous studies investigating overall survival in FL (Table 4) [3,7]. Additional adjustment with FLIPI categories or first-line rituximab only marginally altered these results (data not shown). Starting follow-up at date of venipuncture instead of diagnosis also did not change the results (data not shown). After correcting for performing 54 tests (27 SNPs and two outcomes), the association of rs1801131 (*MTHFR*) with lymphoma progression remained significant (Bonferroni corrected p = 0.01).

**Table 4:** **Relative risk** ^**A**^ **of lymphoma-specific death and lymphoma progression for selected SNPs previously associated with any follicular lymphoma outcome in at least one previous study**

							**Lymphoma-specific death**		**Progression** ^**B**^	
**Chr**	**Gene**	**SNP**	**Position**	**A1**	**A2**	**MAF**	**HR (95% CI)** ^**A**^	**p-value**	**HR (95% CI)** ^**A**^	**p-value**
1	*CD46*	rs2466571	206006669	A	C	0.49	0.98 (0.73; 1.32)	0.90	1.37 (1.10; 1.72)	0.006
1	*CD55*	rs2564978^C^	205561039	T	C	0.31	0.88 (0.62; 1.25)	0.47	1.13 (0.86; 1.49)	0.38
1	*CFH*	rs1065489	194976397	A	C	0.15	0.89 (0.57; 1.39)	0.60	0.92 (0.65; 1.31)	0.66
1	*CFH*	rs1329423^C^	194913010	C	T	0.22	0.94 (0.66; 1.39)	0.81	1.06 (0.79; 1.42)	0.71
1	*CFH*	rs3766404	194918455	G	A	0.16	1.14 (0.77; 1.68)	0.51	1.12 (0.80; 1.55)	0.52
1	*CFHR5*	rs6694672^C^	195212412	G	T	0.09	1.01 (0.61; 1.67)	0.97	1.02 (0.66; 1.57)	0.93
1	*FCGR2A*	rs1801274	159746369	A	G	0.47	0.76 (0.56; 1.03)	0.08	0.86 (0.68; 1.08)	0.19
1	*MTHFR*	rs1801131^C^	11777063	G	T	0.32	0.69 (0.49; 0.97)	0.03	0.59 (0.45; 0.77)	0.0001^E^
1	*SELE*	rs5361	167967684	C	A	0.11	1.04 (0.64; 1.69)	0.89	0.90 (0.61; 1.32)	0.59
2	*IL1RN*	rs454078^C^	113605264	T	A	0.28	0.99 (0.69; 1.42)	0.96	0.94 (0.72; 1.23)	0.66
3	*FTHFD*	rs1127717^C^	127308749	C	T	0.16	0.91 (0.58; 1.43)	0.69	0.98 (0.70; 1.38)	0.92
4	*IL2*	rs2069762^C^	123597430	C	A	0.26	0.63 (0.43; 0.92)	0.02	0.89 (0.69; 1.16)	0.38
4	*IL8*	rs4073^C^	74824888	A	T	0.46	0.93 (0.70; 1.25)	0.64	0.78 (0.62; 0.97)	0.02
4	*IL8*	rs2227307^C^	74825533	G	T	0.45	0.93 (0.69; 1.25)	0.63	0.75 (0.60; 0.94)	0.01
5	*C9*	rs1421094^C^	39391348	A	G	0.37	1.19 (0.88; 1.61)	0.27	1.00 (0.79; 1.27)	0.98
5	*IL12B*	rs3212227^C^	158675528	G	T	0.17	1.21 (0.81; 1.80)	0.36	1.42 (1.03; 1.94)	0.03
6	*C6orf15*	rs6457327^D^	31182009	A	C	0.42	0.90 (0.66; 1.24)	0.53	0.90 (0.71; 1.14)	0.38
6	*IFNGR1*	rs3799488	137561473	G	A	0.12	0.76 (0.46; 1.24)	0.27	1.12 (0.79; 1.57)	0.53
8	*GGH*	rs719235^C^	64114235	A	C	0.32	0.96 (0.69; 1.34)	0.82	1.00 (0.77; 1.29)	0.99
9	*GALNT12*	rs10819377	100643533	A	G	0.46	0.76 (0.56; 1.03)	0.07	0.89 (0.71; 1.12)	0.33
16	*IL4R*	rs1801275	27281901	G	A	0.25	0.91 (0.64; 1.30)	0.62	0.85 (0.65; 1.12)	0.26
22	*MIF*	rs755622^C^	22566392	C	G	0.16	0.76 (0.49; 1.16)	0.20	0.85 (0.61; 1.18)	0.34

^A^Estimated with Hazard ratio, HR, and 95% confidence interval, CI. Adjusted for age at diagnosis, sex and three principal components.

^B^Swedish cases only (n = 231) for time to progression.

^C^Imputed SNP.

^D^rs6457327 has previously been associated with both FL risk and prognosis.

^E^Opposite direction of association to previous study [15].

The minor allele (A1) was investigated for association except for rs2466571 (*CD46*), where the major allele (A2) was used as reference for easier comparison with previous study result.

We finally stratified patients by the number of deleterious/protective alleles, as defined in three previous studies [3,7,15], but found no significant associations with the outcomes in the present study (data not shown). None of the six SNPs previously reported to be associated with *risk* of FL [22-26] were significantly associated with lymphoma-specific death or progression (Table 4 (rs6457327) and Additional file 1: Table S3).

## Discussion

In this GWAS meta-analysis of common genetic variation in FL prognosis in a total of 586 patients, we did not identify any variants associated with lymphoma-specific death at a genome-wide significant level (p < 5.0 ×10^−8^). The strongest association was observed for tightly linked SNPs located on 17q24 near the *ABCA10* and *ABCA6* genes (rs10491178 HR_random_ = 3.17, 95% CI 2.09-4.79, p_random_ = 5.24 ×10^−8^). Investigating previously reported candidate genes, we found further support of a role for rs2466571 in *CD46* in FL progression. Two SNPs in *IL8* (rs4073 and rs2227307) previously linked with overall survival in FL, where here associated with FL progression. We also observed an association with *MTHFR* and FL progression but the direction was opposite of a previous report on overall survival in FL.

### Genome-wide association study and meta-analysis

The increased rate of lymphoma-specific death for carriers of the A allele of rs10491178 in *ABCA10* on 17p24 was consistent in the SCALE and UCSF cohorts, strengthening the notion of a possible causal association with this region. The association was independent of established prognostic risk factors (lactate dehydrogenase levels, performance status and FLIPI). *ABCA10* is clustered among four other members of the ATP binding cassette (ABC) 1 family on 17q24, including *ABCA6* harboring another five linked associated SNP markers. The ABC transporters - a family of proteins responsible for the movement of a wide variety of xenobiotics, lipids and metabolic products across the cell membranes - are implicated in multidrug resistance [39]. They are overexpressed in several tumor types [39], including FL [40]. The exact function of ABCA10 and ABCA6 remains to be elucidated [41], but the ABCA3 transporter, similar in terms of amino acid sequences and structural organization to ABCA10 and ABCA6 [41], has been shown to impede the efficacy of rituximab in aggressive B-cell lymphomas, and vincristine, anthracyclines and etopside in acute myeloid leukemia when expressed in high levels [42]. Interestingly, rs10491178 (C > T) encodes a premature stop codon (Arg (**C**GA) -- > Stop (**T**GA)) in the *ABCA10* transcript (Refseq NM_080282; Ensembl ABCA10-001) [43].

We also looked up SNPs in high LD (r^2^ > 0.8) with rs10491178 for potential regulatory functions in RegulomeDB, and identified another SNP rs113464685 in *ABCA10*, in strong LD (r^2^ = 1.00) with the top SNPs at 17q24, that lies in the binding site of the transcription factor paired box 5 (PAX5). PAX5 has a pivotal regulatory function in B-cell development and its aberrant expression is correlated with aggressive subsets of B-cell NHL [44]. This SNP is thus an alternative candidate to explain the observed association with the region.

The suggestive associations (p ≤ 10^−6^) of rs3131729 in the Dab, reelin signal transducer, homolog 1 (*DAB1*) gene on 1p32-p31, the intergenic SNP rs11932201 on chromosome 4, and rs2250066 in the kallikrein (*KLK*) 11 gene on 19q13, were also consistent in the SCALE and UCSF studies. The role of the DAB1 protein in the developing nervous system is well studied but for other tissues knowledge is scarce [45]. rs11932201 is flanked by two genes (long intergenic non-protein coding RNA 1085 (*LINC01085*) and CPEB2 antisense RNA 1 (*CPEB2-AS1*)) of which little is known. The *KLK* locus on 19q13 harbors genes encoding a family of serine proteases associated with cancer risk and prognosis in several studies [46].

### Investigation of SNPs with reported prognostic impact in FL and established FL risk SNPs

Upon investigating SNPs linked to FL survival in previous studies, we observed a shorter time to lymphoma progression for C compared to A allele carriers at rs2466571 in *CD46* on 1q32 (HR = 1.37, p_trend_ = 0.006), at a nominally significant level. This is consistent with a recent study of 107 FL patients investigating event-free survival in FL (HR = 1.49, p_trend_ < 0.05) [8]. CD46 is a complement inhibitor, protecting the cell against complement-mediated lysis [47]. High expression levels of membrane-bound complement regulatory proteins such as CD46 in tumors has been shown to suppress anti-tumor T-cell responses, and may inhibit anti-tumor therapeutic activity of monoclonal antibodies, including rituximab in B-cell NHL [47].

The minor alleles of two SNPs in *IL8*, rs4073 and rs2227307, on 4q12-q21 were associated with longer time to progression (rs4073 HR_A_ = 0.78, p = 0.02; rs2227307 HR_G_ = 0.75, p = 0.01). Two previous studies reported an association for the same two SNPs with overall survival in FL; Cerhan et al. observed a statistically significant longer overall survival with homozygosity of the same alleles (rs4073 HR_AA_ = 0.47, 95% CI 0.27-0.79, rs2227307 HR_GG_ = 0.53, 95% CI 0.31-0.91) [7], whereas Aschebrook-Kilfoy et al. observed that the opposite alleles were associated with shorter overall survival (rs4073 HR_TT_ = 2.60, 95% CI 1.10-6.15, rs2227307 HR_CC_ = 2.57, 95% CI 1.07-6.17) [3] (Table 1). Time to FL progression was not evaluated in these former investigations. The A allele of rs4073 has been associated with increased production of IL8 [48].

In our study, rs1801131 in *MTHFR* was associated with time to progression and lymphoma-specific death, and rs2069762 in the promoter of *IL2* with lymphoma-specific death. However, these results are conflicting with previous reports of overall survival in FL and are therefore difficult to interpret [7,15]. For rs1801274, located in *FCGR2A* on 1q23, one of the two most studied variants [5-7,10,13,14,16,17], we observed no associations with lymphoma progression or lymphoma-specific death. This is in line with the results of most previous studies, irrespective of how outcome was defined (progression-free [6,10,14,16,17], event-free [5,6] or overall survival [5,7]).

Several SNPs, including rs6457327, rs10484561 and rs2647012 in the HLA class I and II regions on 6p21, are convincingly associated with FL risk [22,24,25], suggesting an important role of this genetic region in the development of FL, and thereby possibly also in FL progression. Indeed, rs6457327 has been associated with FL transformation [4,18] and overall survival [4] in two previous studies (Table 1). However, we did not observe any clear associations for rs6457327, rs10484561 and rs2647012 with FL progression or lymphoma-specific death in our cohort.

### Strengths and weaknesses

The strengths of the present analysis include the population-based design of the two cohorts, the large number of patients compared to previous studies, and the comprehensive evaluation of the impact of genetic variation in FL outcome. Population stratification was adjusted for in the GWAS and we observed little or no evidence of inflation of the statistics (lambda GC_SCALE_ = lambda GC_UCSF_ = 1.00) [49]. In the analysis of candidate gene variants and lymphoma progression or death, adjustment for established risk factors including age, sex, FLIPI categories and first-line rituximab resulted in marginal changes of the point estimates only.

Although candidate gene studies have obvious weaknesses compared with genome-wide studies, a strength include a higher prior probability of true findings, since they are based on a biological understanding of cancer survival pathways [50]. Still, many candidate gene findings cannot be replicated, suggesting some false-positive results in the existing literature [50]. Most of the previous studies were small (N < 150) and the number of SNPs tested were sometimes large (up to 6679 variants), resulting in a considerable risk of chance findings. However, the present study was limited to detect moderate to strong effects (HR >1.5-2.0) for the investigated SNPs with acceptable probability (≥0.80). Hence, chance findings cannot be excluded.

In the study of genetic variation and FL prognosis, different outcome measures have been used, including overall and lymphoma-specific survival and event-free survival. Progression-free survival, defined as the time from study entry to lymphoma progression or all-cause death, is generally recognized as the most valid measure in intervention studies [51]. We argue that lymphoma-specific survival/death may be superior to overall survival (but not to progression-free survival) if the study aims to test an association with lymphoma progression leading to death. In view of the high median age at diagnosis of FL and the often indolent clinical course [1], a relatively large proportion of the patients are expected to die of non-lymphoma related causes, which could dilute associations with lymphoma progression and death if overall survival is used instead. On the other hand, with the use of lymphoma-specific survival some deaths due in part to progression may have been missed potentially leading to a lower specificity. We did not observe similar results for three out of four top genetic variants and lymphoma progression, as with lymphoma-specific death, although this analysis could only be performed in a subset of the patients. Explanations for such a difference could lie in the definitions of these outcomes. While time to progression and progression-free survival primarily reflect the first part of the follow-up period, including mainly time to first-line or second-line treatment, time to lymphoma-specific death reflects a larger part of the follow-up and thus, to a higher degree, treatment response or lack of such. In the future, attempts to validate the current findings, and when comparing results between investigations, the use of similar definitions of outcome will be important for the interpretation.

## Conclusions

In the present GWAS meta-analysis, there was suggestive evidence that inherited polymorphisms in the *ABCA10* or *ABCA6* genes may be associated with risk of lymphoma-specific death. In candidate-gene analysis, an association with lymphoma progression was observed for SNPs in *CD46* and *IL8,* previously linked with lymphoma progression (*CD46*) and overall survival (*IL8*). These findings need further confirmation in other independent populations or in a larger multicenter GWAS.

## Additional file

Additional file 1: Table S1. Top SNPs associated with lymphoma-specific death (prandom<5.0 x10-5) in the meta-analysis of FL patients in SCALE and UCSF (N=586). The minor allele (A1) was investigated for association. A2=major allele, MAF= minor allele frequency, NA= not applicable. **Table S2.** Relative risk of lymphoma-specific and all-cause death in SCALE (N=373), and lymphoma progression in SCALE Sweden (N=231) for the top SNPs in the pooled analysis of SCALE and UCSF. The minor allele was investigated for association. **Table S3.** Relative risk of lymphoma-specific death and lymphoma progression for SNPs previously associated with follicular lymphoma risk. **Figure S1.** Manhattan plot of the -log10(p-values) for the association with lymphoma-specific death in SCALE (A) and UCSF (B), by chromosome and within chromosome location. The top ten SNPs in the meta-analysis are highlighted (light green). The red line marks -log10(5.0 x10-8). **Figure S2.** QQ plot of the observed versus the expected -log(p-values) for the association with lymphoma-specific death in SCALE (A) and UCSF (B). **Figure S3.** Manhattan plot of the -log10(p-values) for the association with lymphoma-specific death in the meta-analysis of SCALE and UCSF. The top ten SNPs are highlighted (light green). The red line marks -log10(5.0 x10-8). **Figure S4.** QQ plot of the observed versus the expected -log(p-values) for the association with lymphoma-specific death in the meta-analysis of SCALE and UCSF. **Figure S5.** Regional association plot of the top locus in the combined analysis on 17q, displaying the p-values of association in SCALE, extent of LD with rs10491178 as reference, relation of SNPs and genes, and estimated recombination rates at each position. **Figure S6.** Estimations of the power to detect associations between selected candidate SNPs and lymphoma-specific death and lymphoma progression, respectively, for different effect sizes and minor allele frequencies (MAF).
