# Supplementary material for: A comprehensive evaluation of the role of genetic variation in follicular lymphoma survival
Source: BMC Med Genet. 2014 Oct 8;15:113. doi: 10.1186/s12881-014-0113-6 (PMC4411784; doi:10.1186/s12881-014-0113-6)

SUPPORTING INFORMATION

Supplement to:

A comprehensive evaluation of the role of genetic variation in follicular lymphoma survival

Fredrik Baecklund et. al. 2014

Correspondence should be addressed to:

Fredrik Baecklund, MD

Unit of Clinical Epidemiology

Dept of Medicine Solna, Karolinska Institutet

Karolinska University Hospital

SE-171 76 Solna, Sweden

E-mail: fredrik.baecklund@ki.se.

Supplementary Table 1 Top SNPs associated with lymphoma-specific death (p_{random}<5.0 x10^{-5}) in the meta-analysis of FL patients in SCALE and UCSF (N=586). The six top SNPs on chromosome 17 and the two top SNPs on chromosome 1 are in LD (in SCALE r^2≥0.96 and r^2=0.7, respectively). The minor allele (A1) was investigated for association. A2=major allele, MAF= minor allele frequency, NA= not applicable

						SCALE		UCSF		Meta-analysis		Heterogeneity				
**CHR**	**SNP**	**Position**	**A1**	**A2**	**MAF**	**HR (95% CI)**	p_{SCALE}	**HR (95% CI)**	p_{UCSF}	**HR (95% CI)**	p_{RANDOM}	p_{HET}	I^2	**Gene**	**Left gene**	**Right gene**
17	rs10491178	64661568	A	G	0.06	3.10 (1.97; 4.89)	1.13E-06	3.50 (1.28; 9.53)	1.36E-02	3.17 (2.09; 4.79)	5.24E-08	0.83	0	ABCA10	ABCA6	LOC100133319
17	rs6502005	64615106	C	A	0.06	3.06 (1.94; 4.84)	1.49E-06	3.79 (1.33; 10.9)	1.20E-02	3.17 (2.09; 4.82)	6.48E-08	0.71	0	ABCA6	ABCA9	ABCA10
17	rs8067965	64629023	G	A	0.06	3.06 (1.94; 4.84)	1.49E-06	3.55 (1.25; 10.0)	1.61E-02	3.14 (2.07; 4.76)	8.12E-08	0.80	0	ABCA6	ABCA9	ABCA10
17	rs7212506	64613313	G	A	0.06	3.06 (1.94; 4.84)	1.49E-06	3.54 (1.25; 10.0)	1.62E-02	3.14 (2.07; 4.76)	8.15E-08	0.80	0	ABCA6	ABCA9	ABCA10
1	rs3131729	57971355	A	G	0.13	2.58 (1.77; 3.76)	9.24E-07	2.00 (0.92; 4.31)	7.58E-02	2.45 (1.75; 3.44)	2.22E-07	0.56	0	DAB1	C8B	LOC729423
17	rs1468514	64608807	A	G	0.06	3.09 (1.91; 5.00)	4.16E-06	3.55 (1.25; 10.0)	1.61E-02	3.17 (2.05; 4.90)	2.24E-07	0.82	0	ABCA6	ABCA9	ABCA10
17	rs2886048	64600537	A	G	0.06	3.09 (1.91; 5.00)	4.16E-06	3.55 (1.25; 10.0)	1.61E-02	3.17 (2.05; 4.90)	2.24E-07	0.82	0	ABCA6	ABCA9	ABCA10
4	rs11932201	14304530	C	A	0.17	2.10 (1.48; 2.97)	2.93E-05	2.50 (1.13; 5.55)	2.29E-02	2.16 (1.57; 2.97)	2.24E-06	0.69	0	NA	LOC152742	LOC441009
1	rs1323822	57999180	A	G	0.11	2.38 (1.60; 3.56)	2.21E-05	1.84 (0.84; 4.04)	1.24E-01	2.26 (1.58; 3.23)	7.76E-06	0.57	0	DAB1	C8B	LOC729423
19	rs2250066	56220931	A	G	0.15	2.07 (1.45; 2.97)	7.31E-05	2.35 (1.01; 5.44)	4.40E-02	2.11 (1.52; 2.94)	9.38E-06	0.79	0	KLK11	KLK10	KLK12
20	rs846222	58658321	A	G	0.20	2.08 (1.44; 2.99)	8.21E-05	1.94 (0.91; 4.12)	8.14E-02	2.05 (1.48; 2.85)	1.74E-05	0.87	0	NA	MTCO2L	CDH4
18	rs2061158	63168592	A	G	0.19	2.00 (1.43; 2.82)	6.27E-05	1.69 (0.84; 3.40)	1.38E-01	1.94 (1.43; 2.63)	2.22E-05	0.67	0	NA	CDH19	DSEL
17	rs8074294	61902137	G	A	0.39	1.82 (1.33; 2.48)	1.54E-04	1.85 (0.95; 3.59)	6.61E-02	1.83 (1.38; 2.42)	2.67E-05	0.97	0	PRKCA	APOH	CACNG5
6	rs10484325	7502047	G	A	0.17	2.14 (1.50; 3.05)	2.80E-05	1.45 (0.59; 3.55)	4.15E-01	2.03 (1.46; 2.82)	2.77E-05	0.43	0	DSP	LOC100131914	C6orf151
9	rs11788432	94828444	G	A	0.29	1.82 (1.30; 2.56)	5.50E-04	2.82 (1.27; 6.26)	1.00E-02	1.95 (1.42; 2.66)	2.93E-05	0.32	0	FGD3	LOC642959	SUSD3
7	rs2060723	131551689	A	G	0.38	1.81 (1.33; 2.46)	1.55E-04	1.71 (0.93; 3.16)	8.13E-02	1.79 (1.36; 2.35)	3.25E-05	0.88	0	PLXNA4	LOC100129705	FLJ40288
7	rs12668564	95278209	G	A	0.21	1.82 (1.30; 2.56)	5.36E-04	2.22 (1.14; 4.32)	1.77E-02	1.90 (1.40; 2.57)	3.31E-05	0.61	0	DYNC1I1	PDK4	SLC25A13
4	rs1868416	59316610	G	A	0.41	1.82 (1.34; 2.49)	1.53E-04	1.66 (0.92; 2.99)	8.72E-02	1.79 (1.36; 2.35)	3.49E-05	0.78	0	NA	SRIL	LPHN3
13	rs9551348	26475083	G	A	0.20	1.82 (1.28; 2.57)	7.72E-04	2.25 (1.15; 4.43)	1.75E-02	1.90 (1.39; 2.59)	4.78E-05	0.58	0	NA	GPR12	LOC100129306

Supplementary Table 2. Relative risk[A] of lymphoma-specific and all-cause death in SCALE (N=373), and lymphoma progression in SCALE Sweden (N=231) for the top SNPs in the pooled analysis of SCALE and UCSF. The minor allele was investigated for association. The minor allele (A1) was investigated for association. A2=major allele, MAF= minor allele frequency, NA= not applicable

						SCALE				SCALE Sweden		
						Lymphoma-specific death		All-cause death		Lymphoma progression		
**CHR**	**SNP**	**Position**	**A1**	**A2**	**MAF**	**HR (95% CI)**	**p**	**HR (95% CI)**	**p**	**HR (95% CI)**	**p**	**Gene**
17	rs10491178	64661568	A	G	0.06	3.10 (1.97; 4.89)	1.13E-06	2.00 (1.35; 2.97)	5.68E-04	1.02 (0.63; 1.65)	9.40E-1	ABCA10
1	rs3131729	57971355	A	G	0.13	2.58 (1.77; 3.76)	9.24E-07	1.86 (1.37; 2.53)	6.46E-05	1.68 (1.20; 2.35)	2.00E-3	DAB1
4	rs11932201	14304530	C	A	0.17	2.10 (1.48; 2.97)	2.93E-05	1.56 (1.17; 2.08)	2.30E-03	1.31 (0.97; 1.75)	7.00E-2	NA
19	rs2250066	56220931	A	G	0.15	2.07 (1.45; 2.97)	7.31E-05	1.50 (1.10; 2.04)	1.05E-02	1.13 (0.82; 1.56)	4.70E-1	KLK11

[A] Estimated with Hazard ratio, HR, and 95% confidence interval, CI. Adjusted for age at diagnosis, sex and three principal components.

Supplementary Table 3. Relative risk[A] of lymphoma-specific death and lymphoma progression for SNPs previously associated with follicular lymphoma risk[B]. The minor allele (A1) was investigated for association. A2=major allele, MAF= minor allele frequency

							Lymphoma-specific death		Progression[C]	
CHR	Gene	SNP	Position	A1	A2	MAF	HR (95% CI)[A]	p	HR (95% CI)[A]	p
2	*BCL2L11*	rs3789068	111625718	A	G	0.45	1.22 (0.91; 1.63)	0.19	0.96 (0.78; 1.19)	0.74
6	HLA II- region	rs2647012	32772436	A	G	0.32	0.70 (0.47; 1.02)	0.06	0.92 (0.69; 1.22)	0.56
6	HLA II- region	rs10484561	32773398	C	A	0.18	0.91 (0.62; 1.33)	0.62	0.77 (0.58; 1.02)	0.07
10	*FAS*	rs4934436	90773300	G	A	0.48	0.84 (0.62; 1.14)	0.27	0.98 (0.78; 1.25)	0.89
13	*TNFSF13B (BAFF)*	rs2582869	107710146	A	G	0.40	1.32 (0.97; 1.79)	0.07	1.08 (0.86; 1.36)	0.51

[A] Estimated with Hazard ratio, HR, and 95% confidence interval, CI. Adjusted for age at diagnosis, sex and three principal components.

[B] rs6457327 has previously been associated with both FL risk and prognosis. The results are shown in Table 4.

[c] Swedish cases only (N=231) for time to progression

Supplementary Figure 1. Manhattan plot of the -log10(p-values) for the association with lymphoma-specific death in SCALE (A) and UCSF (B), by chromosome and within chromosome location. The top ten SNPs in the meta-analysis are highlighted (light green). The red line indicates the cut off level for genome-wide significance -log10(5.0 x10^{-8})

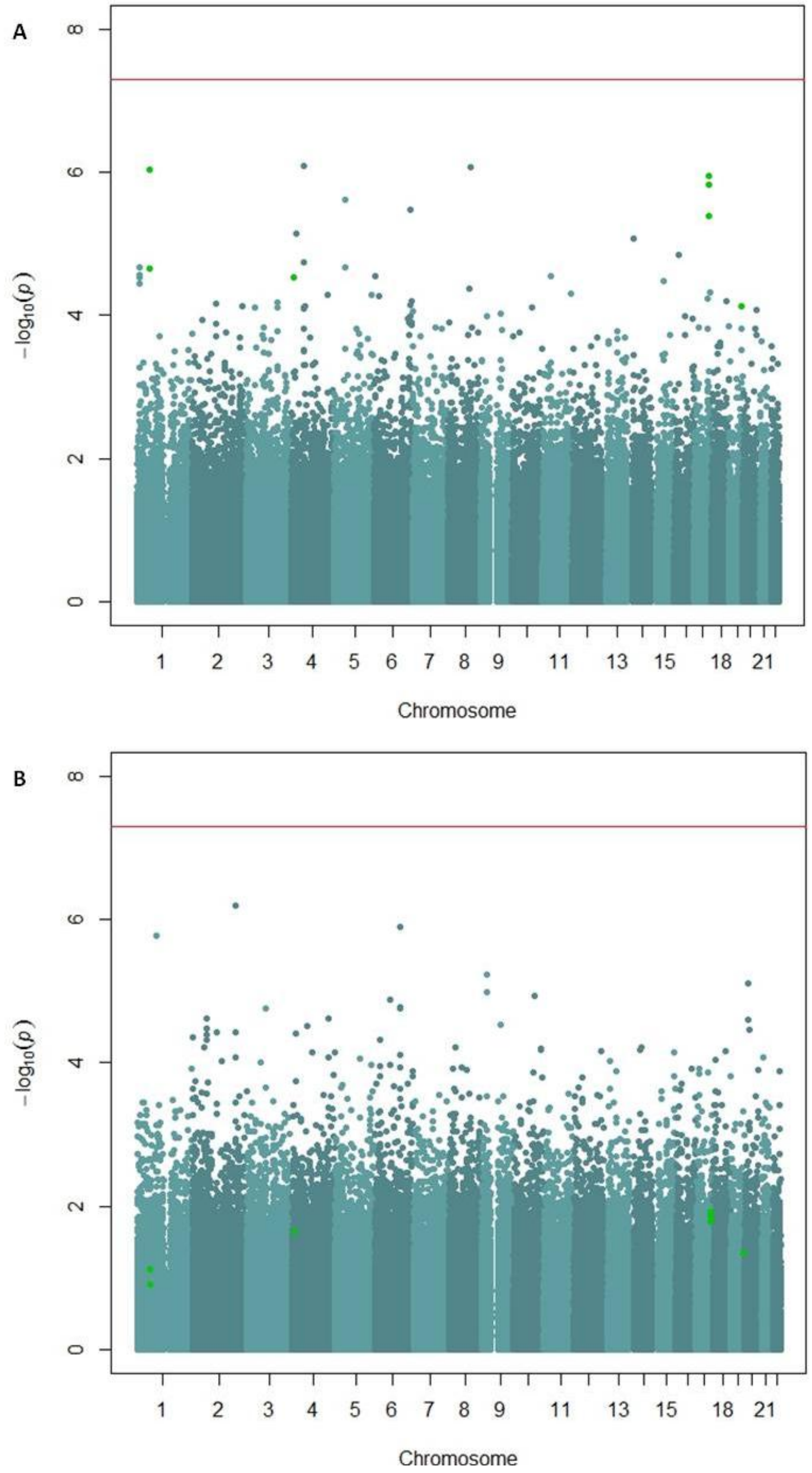

Supplementary Figure 2. QQ plot of the observed versus the expected -log(p-values) for the association with lymphoma-specific death in SCALE (A) and UCSF (B)

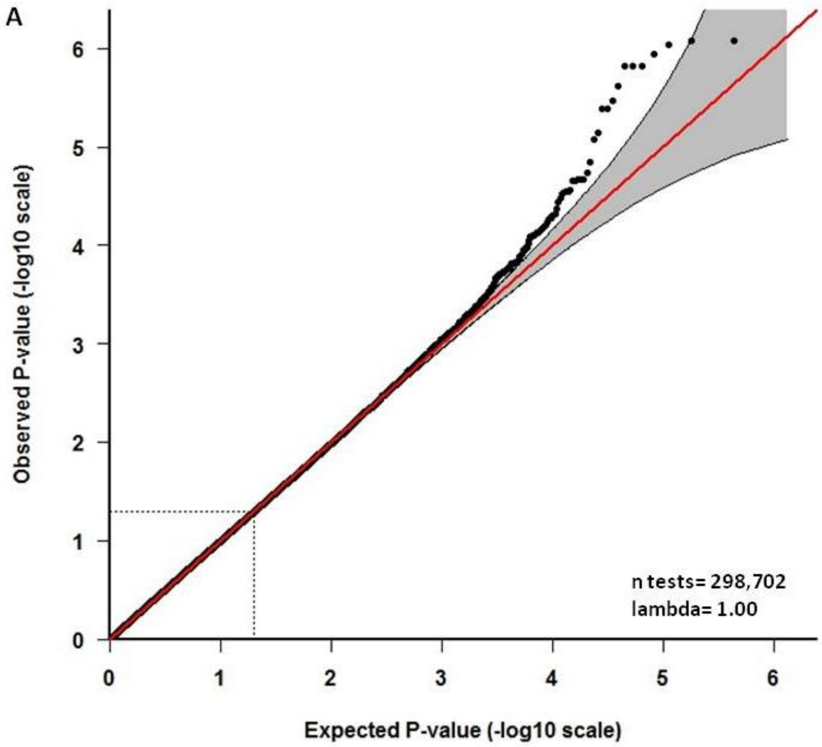

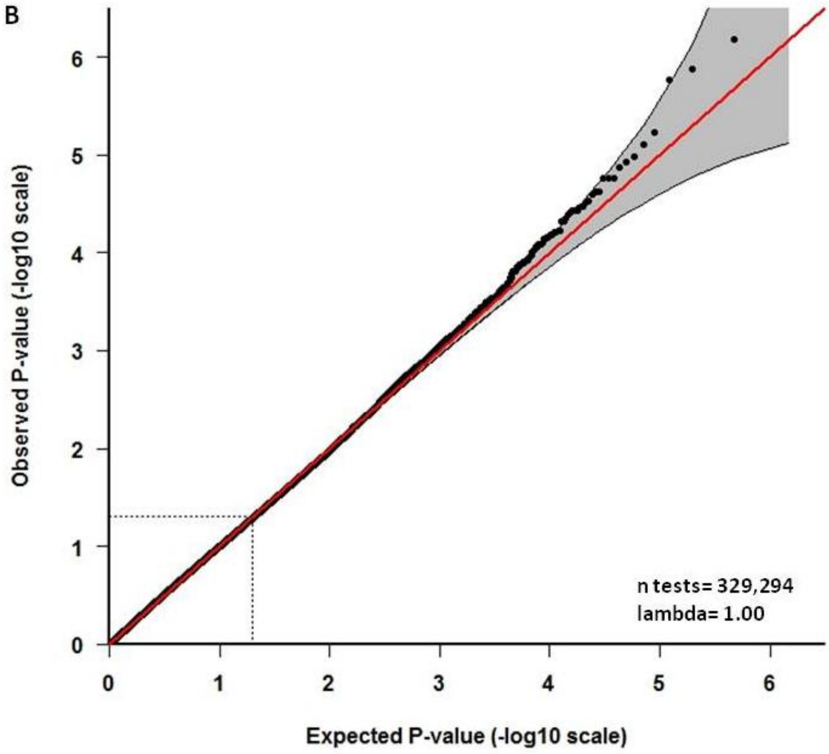

Supplementary Figure 3. Manhattan plot of the -log10(p-values) for the association with lymphoma-specific death in the meta-analysis of SCALE and UCSF. The top ten SNPs ($p_{random} \leq 10^{-6}$) are highlighted (light green). The red line indicates the cut off level for genome-wide significance -log10(5.0 x10^{-8})

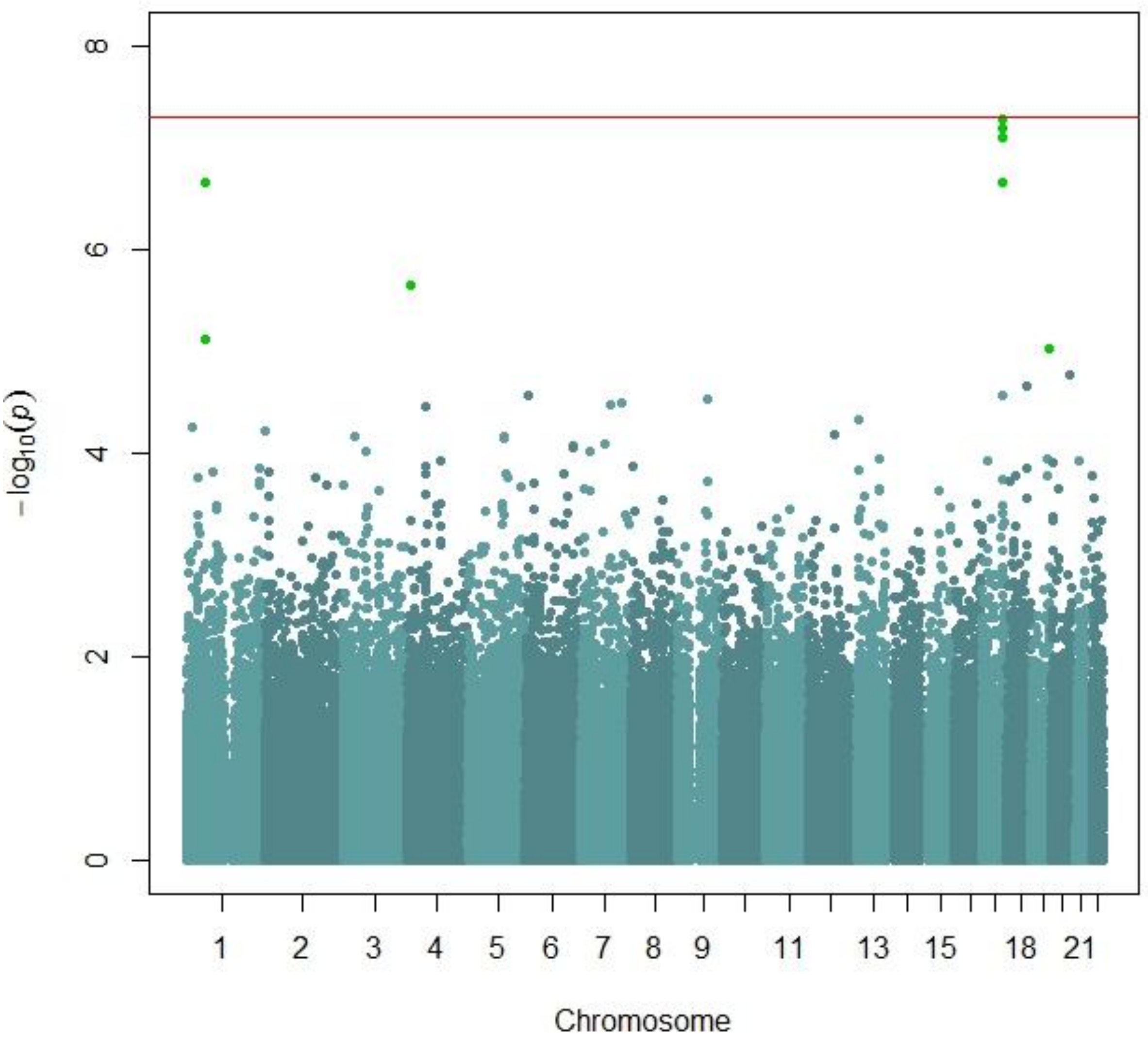

Supplementary Figure 4. QQ plot of the observed versus the expected -log(p-values) for the association with lymphoma-specific death in the meta-analysis of SCALE and UCSF

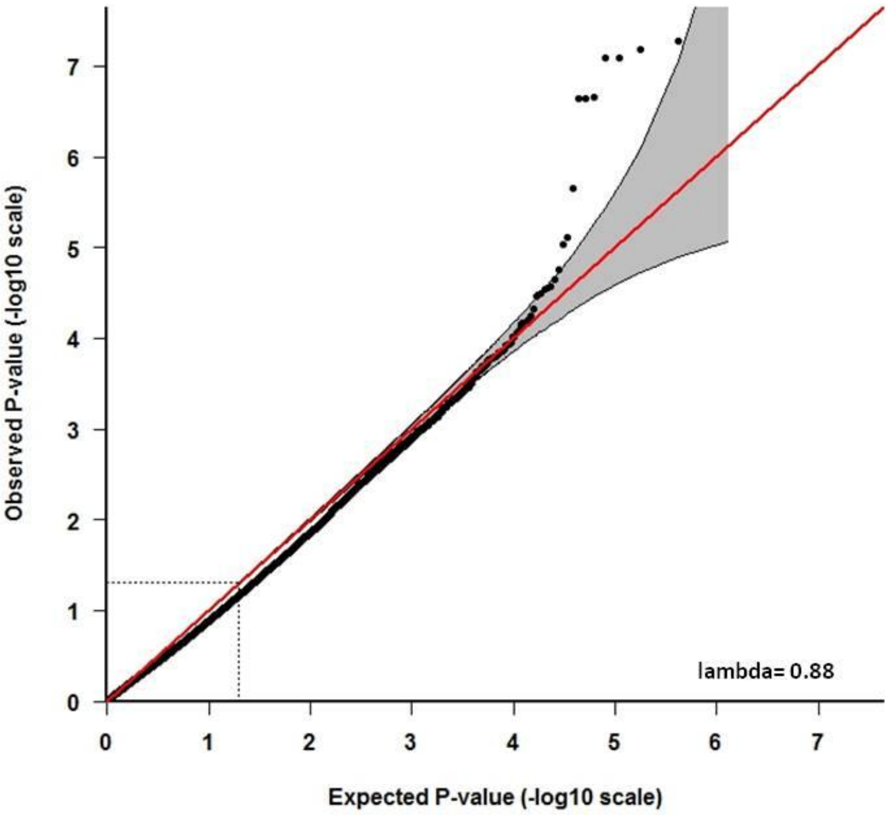

Supplementary Figure 5. Regional association plot of the top locus in the combined analysis on 17q, displaying the p-values of association in SCALE, extent of LD with rs10491178 as reference (using data from 1000 Genomes EUR), relation of SNPs and genes, and estimated recombination rates at each position

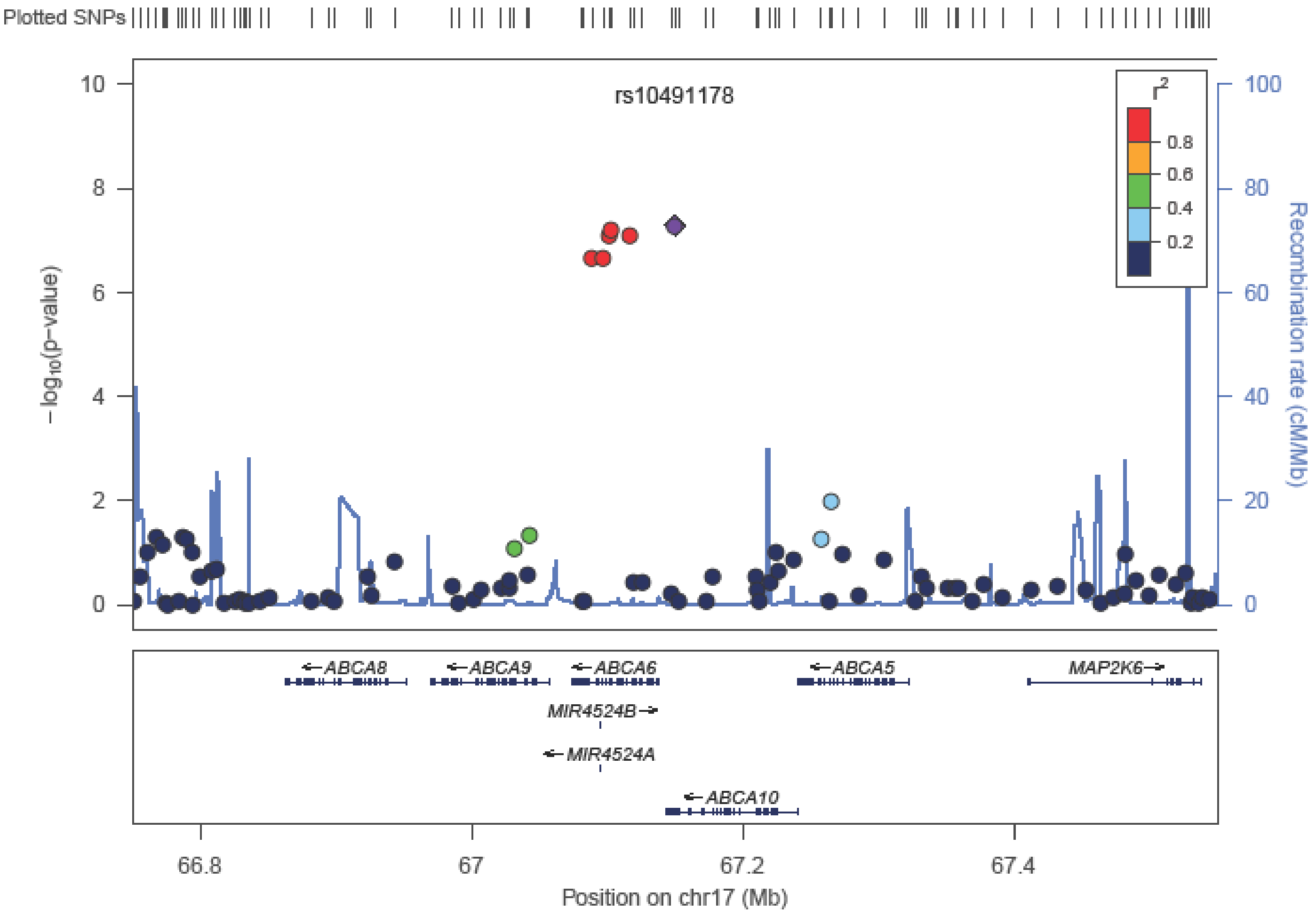

Supplementary Figure 6. Estimations of the power to detect associations between selected candidate SNPs and lymphoma-specific death and lymphoma progression, respectively, for different effect sizes and minor allele frequencies (MAF)

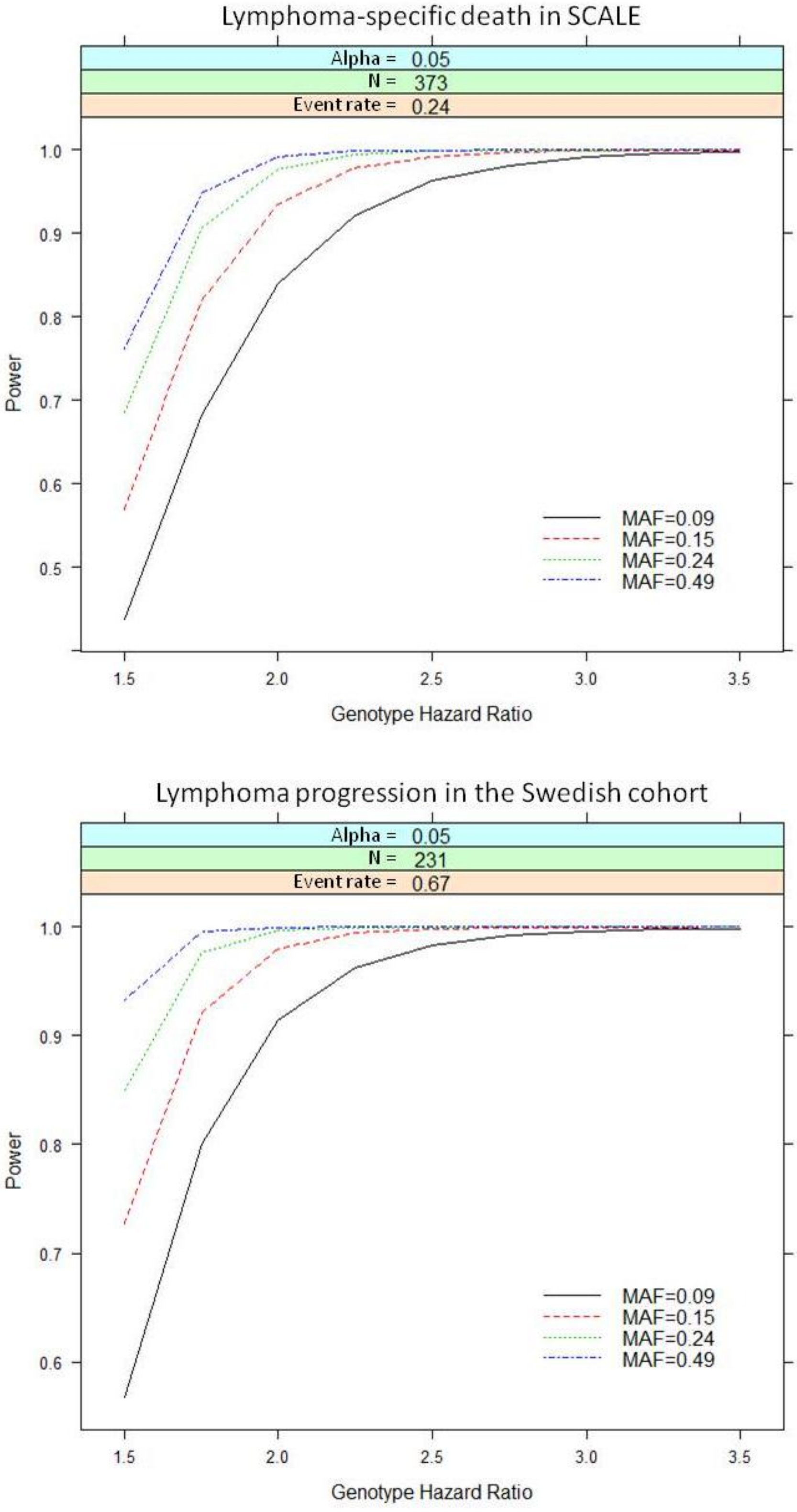

Supplement: Additional file 1: Table S1. — Top SNPs associated with lymphoma-specific death (prandom<5.0 x10^-5^) in the meta-analysis of FL patients in SCALE and UCSF (N=586). The minor allele (A1) was investigated for association. A2=major allele, MAF= minor allele frequency, NA= not applicable. Table S2. Relative risk of lymphoma-specific and all-cause death in SCALE (N=373), and lymphoma progression in SCALE Sweden (N=231) for the top SNPs in the pooled analysis of SCALE and UCSF. The minor allele was investigated for association. Table S3. Relative risk of lymphoma-specific death and lymphoma progression for SNPs previously associated with follicular lymphoma risk. Figure S1. Manhattan plot of the -log10(p-values) for the association with lymphoma-specific death in SCALE (A) and UCSF (B), by chromosome and within chromosome location. The top ten SNPs in the meta-analysis are highlighted (light green). The red line marks -log10(5.0 x10^-8^). Figure S2. QQ plot of the observed versus the expected -log(p-values) for the association with lymphoma-specific death in SCALE (A) and UCSF (B). Figure S3. Manhattan plot of the -log10(p-values) for the association with lymphoma-specific death in the meta-analysis of SCALE and UCSF. The top ten SNPs are highlighted (light green). The red line marks -log10(5.0 x10^-8^). Figure S4. QQ plot of the observed versus the expected -log(p-values) for the association with lymphoma-specific death in the meta-analysis of SCALE and UCSF. Figure S5. Regional association plot of the top locus in the combined analysis on 17q, displaying the p-values of association in SCALE, extent of LD with rs10491178 as reference, relation of SNPs and genes, and estimated recombination rates at each position. Figure S6. Estimations of the power to detect associations between selected candidate SNPs and lymphoma-specific death and lymphoma progression, respectively, for different effect sizes and minor allele frequencies (MAF). [file 12881_2014_113_MOESM1_ESM.pdf]
